# Emotional Competence as a Positive Youth Development Construct: A Conceptual Review

**DOI:** 10.1100/2012/975189

**Published:** 2012-04-26

**Authors:** Patrick S. Y. Lau, Florence K. Y. Wu

**Affiliations:** Department of Educational Psychology, The Chinese University of Hong Kong, Hong Kong

## Abstract

The concept of emotional competence as a positive youth development construct is reviewed in this paper. Differences between emotional intelligence and emotional competence are discussed and an operational definition is adopted. Assessment methods of emotional competence with an emphasis on its quantitative nature are introduced. In the discussion of theories of emotional competence, the functionalist and developmental perspectives and the relationships with positive youth development are highlighted. Possible antecedents, especially the influence of early child-caregiver, and expected outcomes of emotional competence are examined. Practical ways to promote emotional competence among adolescents, particularly the role of parents and teachers, and the future direction of research are also discussed.

## 1. Background

Cognitive intelligence has received much attention as the single most important predictor of human performance [1]. However, the notion of emotional competence is gaining more attention and has been signified as a strong predictor of life success [2]. Emotional competence (EC) can be understood as a group of generic skills that can be applied to many types of emotion-related skills [3]. The ability to identify and discriminate emotions is especially important in youth development [4] and may be influenced by a person's initial orientation to his/her emotion-related problems. When an individual has an ineffective orientation, he/she will try to avoid thoughts and feelings related to the problem [5]. In such a case, he/she may fail to identify emotions and thus be less able to resolve emotional problems in constructive ways and less likely to accept his/her own feelings.

Although references on developing competence related to emotions have been found in both UK and US government reports, the research field is not yet very developed [2]. In the United Kingdom, emphasis on the “well-being” of children has been amplified. The government has set various goals for the educational settings to develop government-approved materials in developing emotional competence in Britain's youth. Government-determined national targets give rise to the need for young people to excel in social and emotional skills. In the United States, the government is equally focused on child well-being, with its “No Child Left Behind” legislation representing a commitment to ensure that all children are provided with effective learning and the opportunity to achieve through development of emotional skills.

The debate in the literature over the terms, “Emotional Intelligence (EI)” or Emotional Competence remains active. Some researchers even deliberately avoid the use of the term, “emotional intelligence” as the distinction between EC and EI is still not clear [4]. Lau [6] articulated the difference between emotional intelligence and emotional competence in his review. The review places the emphasis of EI primarily on in-born ability while the proponents of EC emphasize the skills acquired through cultural and contextual interferences as one develops. In this paper, we take Saarni's [7] “Emotional competence” as the more general and neutral term. We agree with Saarni that the regulation and stabilization of emotions may not assemble general intelligence that is inherited or in-born. Emotional competence can be nurtured and developed as a person grows.

## 2. Definition of Emotional Competence

In the 1920s and 1930s, many psychologists explored emotional intelligence in the arena of “social intelligence” as a single concept [8]. Goleman [9] furthered his research in emotional competencies in relation to two key domain facets: ability and target. Salovey and Mayer [10] first used the term “emotional intelligence” and stated it in four domains: knowing one's emotions, knowing others' emotions, handling one's emotions, and handling others' emotions. Recently, psychologists have been paying attention to the complexity of the construct and describing it in terms of multiple capabilities and competencies [11, 12]. The multiplicity and integration of the concepts provide a more comprehensive framework for investigating emotional competence. Emotional competence is understood as the capabilities that are used as predictors of performance and effectiveness in management and leadership [13]. Boyatzis et al. [13] offer a descriptive definition of emotional competence that “a person demonstrates the competencies that constitute self-awareness, self-management, social awareness, and social skills at appropriate times and ways in sufficient frequency to be effective in the situation” (p.3).

Emotional intelligence is a “convenient phrase” that focuses on human talent [13]. However, EI has been challenged for having too broad a definition of all positive personality traits that result in positive outcomes. The famous model proposed by Goleman [8, 9] does not mention the ways to distinguish the level of a person's EI. Zeidner et al. [14] state that the definition of EI may be mixed with social intelligence (SI) as both constructs measure the individual differences relative to how much an individual exhibits traits. In their review, Zeidner et al. [14] point out that EI is mostly defined as a stable quality of the individual. However, most of the adaptive responses to different emotional circumstances are dynamic and situation dependent. In this sense, using the causal model among the constructs of EI is meaningless. Hence, it would be better to define EI as a set of supporting adaptations of emotional skills, which the proponents of EC emphasize, that would form the causal, positive outcomes [15].

Carroll [16] comments that the EI construct lacks the comprehensive models established for conventional mental abilities and Zeidner et al. [14] echo that it is difficult to differentiate EI from multiple constructs due to the difficulty in conceptualization. The developmental psychologists find that the term “intelligence” focuses more on the “mental ability” [17] and characteristics of the person [18] without the notion of contextual influences on the individual. Most recently, there has developed a separate strand of research, focusing on the conceptions, awareness, understanding, and applications of the emotions in social interactions [3]. EI can thus be viewed as a snapshot of emotional competencies and the term “emotional competence” is adopted in a more neutral way [7]. The word “competence” signifies the generalization of the most emotive situations [14]. As such, we agree with Ciarrochi and Scott [4] that emotional competence includes the ability to identify emotions and an individual difference in how effectively people deal with emotions and emotionally charged problems.

The ideations between the constructs of EC and EI are substantially overlapping yet conceptually different. The trait EI, one of the two predominant perspectives in conceptualizing EI, sees EI as a kind of competence that indicates “one's ability to succeed in coping with environment demands and pressures” [11, p.14]. Trait EI shares some commonalities with EC, in that it conceptualizes “intelligence” as “competences” and qualities of individuals to aid them to utilize the competence in real-life situations [19]. The major differentiation between EC and EI is the conceptualization of learning the emotions. The essence of EI is more on an individual's traits and personality in response to the emotion displayed. Yet the proponents of EC are prone to the strand of developmental approach. The competence is gained through development of skills acquired by context- and cultural-related experiences with others. Children can learn specific emotional behaviors for their culture as a result of social interaction. Emotional competence is transactional within self and between self and others, yet EI is less transactional as the model is centered within the individual [20]. Saarni [21] furthers this distinction in her recent review, stating that there are three significant conceptual differences between EI and EC, which are as follows: (1) EC is seen as a set of developed skills; (2) individuals that are emotionally competent are reacting to the emotion-eliciting environments with skills whereas emotionally intelligent individuals are responding with traits residing within those individuals; (3) third is the contribution of personal integrity to mature, emotionally competent functioning. Therefore, we take EC as the discussion focus as we believe that the focus of growth should be on how much the individuals apply their potential and skills in life contexts, rather than emphasizing internal ability in dealing with emotion-laden situations.

Furthermore, the word “competence” could also be referred to a person's mastery of some skills in the traditional western psychology [22]. Based on this tradition, Saarni [7] proposed eight skills as the components of emotional competence to handle emotion-eliciting social transactions. In brief, these eight skills include (1) being aware of one's own emotions, (2) discerning and understanding others' emotions, (3) using the vocabulary of emotion and expressions, (4) having the capacity for empathic involvement, (5) differentiating internal, subjective emotional experience from external, emotional expression, (6) coping adaptively with aversive emotions and distressing circumstances, (7) being aware of emotional communication within relationships, and (8) possessing the capacity for emotional self efficacy. Lau [6] classified these eight skills into two broad domains: (1) skills and (2) form the perceptual domain and the others form the behavioral domain. Integrating the key concepts of emotional competence in the literature, Lau [6] then summarized three major components of emotional competence as its operational definition. These three components include the skills for identifying personal feelings and those of others, the skills for communicating emotions with others, and the skills for coping with negative emotions and set-backs. In this paper, this operational definition of emotional competence is adopted.

## 3. Theories of Emotional Competence

Theories of the emotional competence construct are crucial to understanding the application of skills of the individuals to the emotion-laden environments [23]. There are two dimensions to infer the theories of emotional competence: (1) the construct related to the socialization in respect of functionalist and developmental perspectives and (2) the relationship between the construct and positive youth development.

Lazarus [24] and Campos et al. [25] first proposed to view competences related to emotions from a functionalist point of view, and Saarni [21, 23] advanced this perspective from both the functionalist and developmental angles. In the functionalist perspective, the purpose of responding the stimulations of significant events or situations is stressed. The emotional competence can be developed in response to the dynamic interactions with significant others in the environment. An individual gains the interpretation of different emotions by the environmental and interpersonal stimuli as he or she moves through different developmental stages. In line with the functionalist perspective, Saarni [23] discussed EC under the assumption that emotional development would be affected by the interactions among human beings and with the “ethno-psychological ecology”, that is, the culture and social world. The skills in managing and regulating emotions can be acquired through learning and the interpretation of the emotion-eliciting environment with the emphasis on the interpersonal and social interactions within it. Although the competence can be gained developmentally, Saarni [18, 21, 23] remarks that the acquisition of emotional competence would not be sequential. Each skill “reciprocally influences the differentiation of the other skills” in human development [23, p.30].

The second dimension in understanding the theories of the construct is in relation to positive youth development. The perception of the problems generated in the emotion-laden contexts exerts influences on adolescents' emotional well-being. Concerning the well-being of the adolescents, emotional problems were found to be one of the key competence variables in a large cross-sectional study by Ciarrochi et al. [26]. Ineffective orientation to emotion-related problems is related to the difficulty in indentifying the emotions. The individuals would then turn to destructive forms of emotional management, such as alcohol abuse [27]. Ciarrochi and Scott [4] administered a longitudinal study to investigate causal relations and the link between emotional competence and well-being. They found that people with effective problem orientation were less likely to experience depression, anxiety, and stress and were more likely to experience positive moods. Catalano et al. [28] state that the enhancement of competence can help prevent other negative outcomes and is indicative of positive youth development.

In her review of the influence of the emotional competence in teaching and learning, Garner [3] articulated the theories derived from psychology and education that affect the development of emotional competence in adolescents. The theories denote the relationship between the positive and stable emotions and academic performance in schools. As shown in past studies, Garner [3] agreed that adolescents with better managed emotions would perform, both academically and socially, better in schools.

Under the influence of globalization, adolescents are exposed to divergence of their own culture and other cultures. As school-aged children and adolescents are experiencing the trials of understanding emotions and emotional changes [3], the intention of increasing the awareness of the consideration of the cultural norms and the social partners, or “audience” as Saarni [7] claims, becomes the priority. Gross and Levenson [29] echo this priority with reference to the emotional display rule that would help adolescents to identify the socially and culturally unaccepted emotions. The knowledge of the cultural rule is transmitted by the emotion-eliciting situations in the adolescents' culture. As the learning process of emotions is procedural [21], rehearsals of responding to the social contexts would contribute to one's emotional competence.

## 4. Assessment of Emotional Competence

There is a considerable number of assessment tools for EI developed for measuring emotion-related competencies. The measurements originally developed for EI have been commonly used in studying EC, reflecting that these measurements are compatible for assessing EC. Although there are two approaches for assessing EC, namely, quantitative and qualitative methods, the methods generally used are quantitative in nature.

A number of assessment instruments were developed in the past decades and have provided valuable information on social-emotional behaviors in young children [30]. Nevertheless, there are two concerns in using the assessment instruments of EC. The first is that there are few measures available to assess the EC of adolescents [31]. Consistent findings appear in different reviews [30, 32] that few measures are found to be relevant to assess the EC of preschool and young children. The second concern is the speculation regarding the psychometric properties of the related instruments or inventories [2, 33]. Although Bar-On Emotional Quotient Inventory (Bar-On EQ-i) [11] is a popular and commonly used assessment instrument for measuring EC, its validity is frequently challenged and criticized [34].

In addition, self-perception reports are commonly used to report the scale of emotion competence. Some researchers may challenge the format of the measurement. Austin et al. [35] doubt the consistency of the factor structure of the Schutte Self Report Inventory (SSRI) [33] although the internal reliability for overall construct of the scale is good. Most of the measurements of the emotional competence share the basis of self-report measures [1]. It is possible that respondents may inflate their ratings as a result of social desirability [36]. The intention to assess the respondent's tendency of unconscious self-deception should be emphasized in these self-report measures. As such, further development on the consistency of the self-perception reports should be considered.

Despite the inadequate psychometric properties of the Bar-On EQ-i, Saarni [23] commented that this inventory came closest to the construct of EC in providing “a combined social-emotional and personality attribute assessment of children's self-reported emotion-related functioning” (p. 18). In order to supplement the quantitative data obtained by using Bar-On EQ-i in assessing EC, Saarni [23] proposed a series of qualitative methods, such as interviewing the subjects about their emotional experiences, asking the parents and teachers of the subjects to systematically rate and describe the subjects' emotions, observing them directly in emotion-eliciting situations. Thus, combining both quantitative and qualitative methods may be of greater value and should be considered in assessing EC.

## 5. Possible Antecedents of Emotional Competence

The social environment influences the emotional development of the individual. Using attachment theory, Harris [37] and Colle and Del Giudice [38] agree that attachment status and early child-caregiver relationships contribute to children's understanding of emotions and affect children's emotional functioning at all levels [39]. The different attachment patterns of the individuals might have impact on the development of emotional competence from infancy to adulthood. The secure milieu provided by the caregivers, with their openness of expressivity and coregulation efforts, enhances the internalization of effective emotion regulation of children. Hence, children grow in a more stable emotional state and with higher tendency to engage in active problem solving and coping. In contrast, if the children's emotions are not attended with support and care, hyperactivating and deactivating styles of coping strategies will be developed [38]. Hyperactivating children would regulate emotions ineffectively and feel helpless as they fear losing the attention and care of the caregivers. This may hamper the empathic connection with others and the children may have difficulty in acquiring the social skills and emotional communicative strategies for friendship development [23]. Children with deactivating style will avoid expressing their emotions, especially negative emotions and eventually become less aware of their own feelings and emotions [38]. This kind of suppression lasts into adulthood. In the attachment interview of Roisman et al. [40], an increase of electrodermal response, interpreted as high arousal of suppressed emotions, was found in adult participants who lived in a rare care-giving milieu.

Scharfe [41] suggests that the maternal expressivity influences children's capacity of expressing emotions. The role of secure caregivers (usually maternal care) and the sense of security are highlighted in the research conducted by Colle and Del Giudice [38]. Secure children were found to be more capable of regulating their emotions and maintaining organized behaviors during the times of emotional arousals and were showing higher tolerance to frustrations than those children of dismissed and disorganized attachment style. It seems that the sense of security facilitates children to come up with reflective and effective coping strategies that may benefit in managing the negative emotions in social contexts when they grow older [38]. The related literature emphasizes the maternal role in influencing the development of the emotional competence of children.

In line with the recent investigations and findings on the genetic factors contributing to the development of EI, Cassidy [42] suggests that children's temperament, which is found to be genetically inherited, would influence the development and regulation of emotions. The mental status and ability of the individuals exert influence over the understanding emotions too. McAlpine et al. [43] indicate in their research that individuals with mental disabilities like attention-deficit hyperactivity disorder (ADHD) or learning difficulties are less proficient in identifying and understanding emotions.

## 6. Emotional Competence and Adolescent Developmental Outcomes

There is a large number of empirical studies on the expected developmental outcomes of emotional competence among adolescents (e.g., [7, 44, 45]) with special references on the three intriguing consequences: skills in managing one's emotions, a sense of subjective well-being, and adaptive resilience [18].

Colle and Del Giudice [38] point out that the development of emotional competence reaches its critical phase in middle childhood, which would be the time for children to gain understanding of complex emotions and employ emotion regulation strategies. In middle childhood, children start to experience the complexity of the human world and learn how to cope with these situations. Regulating and controlling oneself becomes an essential ability in the context of social life for children at this crucial stage. By adolescence, young people start to become aware of the variations in emotion-evocative situations and try to respond to these changing contexts with proper expressions. Adolescents learn how to develop socially desirable coping strategies with increased maturity and broad exposure of social interactions. Sufficient provision of training on emotional competence to cope effectively with stressful life events is indispensable and beneficial for adolescents during this turbulent life stage.

Although their well-being has been found to decrease in early to middle adolescence and reach its lowest point at age 16 [46], emotional competence is generally hypothesized to be a good predictor of one's sense of subjective well-being [19]. There is an assumption that emotionally competent individuals will have richer sense of subjective well-being. Zeidner and Olnick-Shemesh [19] summarize four reasons for this assumption. First, emotionally competent individuals are more aware of their emotions and more able to regulate them, which will contribute to experience higher levels of well-being. Second, the individuals with emotional competence are assumed to have richer social connections and are able to demonstrate better coping strategies. Third, with more accurate interpretation of the information yielded by the emotions and the environment, individuals with emotional competence can sustain a better sense of well-being. Fourth, provided that those with emotional competence would have the propensity to experience more positive affects, individuals are more prone to a richer sense of subjective well-being.

Having an opportunity to be educated and being young are contributors to subjective well-being [47]. In the educational settings, school success and academic achievement are crucial to adolescent development. Parker et al. [48] reviewed the association between emotional competence and academic achievement of adolescents and conducted a research investigating the relationship between the two. Emotional competence was found to be a significant predictor of academic success for students of all grades without any gender differences [2, 48]. The result is consistent with other studies [49, 50] stating that students with better academic achievements have better management of emotional dimensions including interpersonal, intrapersonal, adaptability, and stress management. However, the directionality works both ways: students with better emotional competence may perform better academically.

Given that the traditional constructs of intelligence do not predict life success [51], the importance of prediction by emotional competence is demonstrated. In predicting life success or life satisfaction of school children and adolescents, adolescents who are more emotionally competent are found less aggressive [52] and less likely to have had unauthorized absence from school [53]. There is also evidence that EC moderates the link between stress and mental health, hopelessness, and suicidal ideation [54].

The term resilience denotes the “defensive connotations” and assumes that the danger or distress must be overcome by personal qualities [55]. Saarni [18] explains these personal qualities as individuals behave with emotional competence—using effective coping strategies and regulating the stressor-eliciting emotions. In their research, Murphy and Moriarty [56] found that if children could behave emotional-competently when they were exposed to stressors that were within their coping capacity, they were more likely to develop new coping strategies and thus the ability to overcome future stressors would be increased. In other words, adaptive resilience could be a consequence of the development of emotional competence.

## 7. Promotions of Emotional Competence among Adolescents

In promoting emotional competence among adolescents, three practical methods are introduced in this review: (1) provision of the platform for discussing emotions, (2) modeling from significant others and the role of family, and (3) scaffolding provided by school-based interventions.

Clore et al. [57] suggest that most emotion processes operate without consciousness. With the provision of a platform for the discussion about emotions, adolescents can gain knowledge in expressing emotions appropriately. The platform is well situated in familial and school settings. If we take the hypothesis of emotional competence as being related to the development of actual skills and the construct is more prone to the individual's perceptions, emotional competence can be taught within social contexts such as family and school settings. While the competence of emotion is the accumulation and understanding of life experiences within the environment [2, 58], the acquisition of EC should involve the teaching of social skills and emotion knowledge [59]. In the process of teaching, the social information processing model is usually adopted to teach children and adolescents the interpersonal cognition (the interpretation of the social interactions among peers) and intrapersonal cognition (the application of actual social skills through the conceptions of their emotions) [60]. Therefore, integrating these concepts and the model in the school curriculum assists the promotion of emotional competence in adolescents. A simple yet logical assumption follows: if emotional skills are taught through a curricular approach in schools, the emotional competence of adolescents will be increased.

In order to assist adolescents to enhance the sensitivity of the learning of emotions, parents can perform as role models to articulate the learning experiences in these situations in their daily life contexts. Saarni [23] emphasizes that the emotion socialization processes of the families of origin are crucial to children's development. If the child is living under a secure and supportive family, the child may experience diverse emotions in a safe and predictable place [61], enabling the child to learn effective coping strategies and be more prosocial. Moreover, emotional management of children is facilitated by a parent who “sympathetically hears what the child has to say, provides reasoned alternatives” [23, page 28]. The supportive parent would act as a good emotion coach to help the child regulate emotional challenges and arousal. In Chinese societies, however, children are socialized and taught to control and suppress emotions within the family [6, 62]. Lau [6] emphasizes that this traditional and patriarchal familial teaching may inhibit the expression of emotions of Chinese children. The difficulty in modulating the emotions leads the Chinese to display more emotional problems when compared with their western counterparts. In addition, the patriarchal culture of Chinese families also affects girls' expressivity of emotions. Girls are perceived as more verbally and facially expressive than boys. However, being influenced by the traditional teaching, Chinese girls are more passive and less willing to exhibit their emotions to others. Lau [6], therefore, suggests that the gender differences should be taken consideration when designing curricula for youth development.

The importance of promoting emotional education is questioned in the educational settings and the response of the educators to the importance of emotional education is mixed. Some claim that emotional education is “the missing piece” [63], yet some teachers may view it with skepticism as they believe that academic achievement is more important. However, as indicated by substantial literature, there is a direct link between the emotional status of the students and their performance in tests and examinations: increased anxiety and stress equates with poorer performance in these areas. Emotional stability indicates the wide array of expected and favorable outcomes. Emotional competence is seen as the knowledge about ourselves and others. It is also of the prime indicator of academic success [64] and this helps to have the capacity to solve problems adaptively, which is the crucial foundation for academic learning. Emotional competence of adolescents could be promoted by school-based intervention programs. The education can be carried out in diverse formats, such as classroom instruction, extra-curricular activities, or curricular-based programs. The emotional education programs aim at promoting skills “to listen or focus, to feel committed and responsible for work, to rein impulses, and to cope with upsetting events” [11, p.222]. This scaffolding provided by school-based programs becomes an important factor for developing students' emotional competence.

## 8. Concluding Thoughts with Future Research Directions

In reviewing the literature, there are constant, overlapping ideations and ambiguity of conceptual formulations of the constructs, EI and EC. Many researchers have addressed the issue and attempted to differentiate these two; yet overlaps still exist. Therefore, conceptual clarification of the two constructs should be emphasized. The validity of the two constructs is challenged and criticized as discussed in the preceding text. Additional studies are needed to validate the uniqueness of both EI and EC.

Much of the research output is investigated in a cross-sectional nature [2, 3, 58]; more longitudinal investigations are expected to inquire into the influences of emotional competence at different time points and stages of life. Different methodological techniques should be adopted in the future investigations on emotional competence. In addition to the traditional and static investigation methods by the quantitative approach, multiple modes of portrayal of emotions and other qualitative methods, such as interviews and observations, can be adopted, as emotions are dynamic and fluid in nature.

The influence of school and family is highlighted in the review, yet the role of personnel, like the influence of teachers and parents, needs further exploration. Researchers are showing interest in inquiring into the teachers' or parents' understanding of the students' emotions in relation to adolescents' development of emotional competence in the socialization of emotions [3, 65]. However, the idea of teachers and parents as agents of emotion socialization has received limited research attention. The work in this arena is still in its infancy and requires further investigation.
